# Colocalization of cerebral iron with Amyloid beta in Mild Cognitive Impairment

**DOI:** 10.1038/srep35514

**Published:** 2016-10-17

**Authors:** J. M. G. van Bergen, X. Li, J. Hua, S. J. Schreiner, S. C. Steininger, F. C. Quevenco, M. Wyss, A. F. Gietl, V. Treyer, S. E. Leh, F. Buck, R. M. Nitsch, K. P. Pruessmann, P. C. M. van Zijl, C. Hock, P. G. Unschuld

**Affiliations:** 1 Institute for Regenerative Medicine, University of Zürich, Switzerland; 2 The Russell H. Morgan Department of Radiology and Radiological Science, Division of MR Research, The Johns Hopkins University School of Medicine, Baltimore, Maryland, USA; 3 F.M. Kirby Research Center for Functional Brain Imaging, Kennedy Krieger Institute, Baltimore, Maryland, USA; 4 Hospital for Psychogeriatric Medicine, University of Zürich, Switzerland; 5 Institute for Biomedical Engineering, University of Zürich and ETH Zürich, Zürich, Switzerland; 6 Department of Nuclear Medicine, University Hospital Zürich and University of Zürich, Zürich, Switzerland

## Abstract

Quantitative Susceptibility Mapping (QSM) MRI at 7 Tesla and 11-Carbon Pittsburgh-Compound-B PET were used for investigating the relationship between brain iron and Amyloid beta (Aβ) plaque-load in a context of increased risk for Alzheimer's disease (AD), as reflected by the Apolipoprotein E **ε**4 (APOE-e4) allele and mild cognitive impairment (MCI) in elderly subjects. Carriers of APOE-e4 with normal cognition had higher cortical Aβ-plaque-load than non-carriers. In MCI an association between APOE-e4 and higher Aβ-plaque-load was observable both for cortical and subcortical brain-regions. APOE-e4 and MCI was also associated with higher cortical iron. Moreover, cerebral iron significantly affected functional coupling, and was furthermore associated with increased Aβ-plaque-load (R^2^-adjusted = 0.80, p < 0.001) and APOE-e4 carrier status (p < 0.001) in MCI. This study confirms earlier reports on an association between increased brain iron-burden and risk for neurocognitive dysfunction due to AD, and indicates that disease-progression is conferred by spatial colocalization of brain iron deposits with Aβ-plaques.

Alzheimer's disease (AD) is the most frequent cause of dementia and significantly increased risk for AD-dementia is associated with advanced age, mild cognitive impairment (MCI) and carrier-status of the Apolipoprotein E **ε**4 allele (APOE-e4)^1^,^2^. Neuropathological hallmarks of AD include both intracellular pathological neurofibrils as well as extracellular accumulation of Amyloid beta (Aβ) plaques^3^,^4^. Particularly the accumulation of Aβ-plaques is considered to potentially represent preclinical disease stages^5^,^6^,^7^. The APOE-e4 allele has been shown to be closely associated with the extent of cerebral Aβ-plaque-load^8^ and both exert interactive effects on cognitive decline^9^. Recent data demonstrate that the risk for developing AD-dementia, as conferred by APOE-e4 carrier status, is closely linked to cerebral iron-burden, implicating potential benefit of therapeutic strategies aimed at lowering brain iron in this patient population^10^,^11^.

Accumulation of iron in the human brain is a characteristic finding in several neurodegenerative disorders^12^,^13^ and has been reported for AD both in post-mortem studies^14^,^15^,^16^ as well as *in vivo* by using magnetic resonance imaging (MRI)^17^,^18^. In its normal function, amyloid precursor protein (APP) facilitates iron transport outside the cell^19^ In mammalian cell cultures iron has been demonstrated to interfere in the aggregation of Aβ and thus may significantly promote Aβ neurotoxicity in AD^20^. Additionally, local iron deposits are considered to reflect mitochondrial dysfunction^21^ and abnormal microglial activation^22^ in a context of pathological brain change taking place in AD. Recent developments on quantitative susceptibility mapping (QSM) techniques^23^,^24^,^25^,^26^ have made it possible to directly map brain tissue magnetic susceptibility, which has been shown to correlate well with tissue iron concentration in cerebral gray matter^24^,^27^,^28^,^29^.

Positron Emission Tomography (PET) for measuring cerebral Aβ-plaque-load has been combined with functional MRI (fMRI) at rest for inferring on functional network integrity, in several studies so far that included both cognitively normal populations of elderly subjects, as well as individuals with MCI and manifest AD-dementia^30^,^31^,^32^. Interestingly, there is a significant overlap between brain regions characterized by altered functional connectivity and localization of AD-pathology^33^. This overlap particularly affects structures connected with the medial prefrontal cortex (MPFC), which is a central hub within the default-mode network (DMN) and thus is considered to have spatially specific effects on neuronal functionality, as reflected by downstream memory deficits^34^,^35^,^36^,^37^. The application of MRI at ultra-high field strength of 7 Tesla (7T) inherently increases signal to noise ratios (SNR) in QSM and BOLD fMRI, due to the linear relationship between susceptibility induced field shift and field strength, and supralinear relationship between BOLD contrast and field strength, respectively, allowing for detecting subtle changes in the brain with relatively small sample size^25^,^38^.

At this point several neuropathological and neuroimaging studies have demonstrated the close relationship between increased risk for AD and prevalence of MCI, APOE-e4 carrier status, Aβ-plaque-load and altered functional connectivity^39^, and also an association between APOE-e4 carrier status and increased brain iron load^10^. However, to our knowledge there are no studies published on the impact of brain iron load on functional brain network integrity and prevalence of regional Aβ-plaque-load in subjects at risk for AD. As the extent of regional Aβ-plaque-load may be estimated by applying radioactive tracers such as 11C-Pittsburgh Compound B for PET (PiB-PET)^40^,^41^ and cerebral iron can be measured by QSM^29^, the combination of QSM with PiB-PET can be used to infer on Aβ-plaque related iron load. Thus, for the current study the following questions were investigated in a study population of elderly subjects with normal cognitive performance and MCI:

- To investigate a potential relationship between increased AD-risk, as reflected by MCI and APOE-e4 carrier-status, with cerebral iron-burden, as measured by whole-brain QSM at ultra-high field strength of 7T.
- To estimate combined effects of MCI and increased cerebral iron-load on MPFC-coupling by resting state BOLD fMRI.
- To characterize the relationship between iron-load and Aβ-plaque density in brain regions with altered MPFC-coupling by whole-brain QSM and 11C-PiB-PET data.

## Methods

### Participants

37 study participants aged between 62 and 89 years (22 cognitively normal, 15 MCI) without evidence of significant medical illness, were recruited as part of an ongoing study at our hospital. The study was conducted in accordance with good clinical practice guidelines issued by the local ethics committee (Kantonale Ethikkommission Zürich), as well as with the declaration of Helsinki. All procedures were approved by the Kantonale Ethikkommission Zürich. Written informed consent was obtained from all participants before inclusion in the study.

All participants received psychiatric examination and neuropsychological testing during screening for eligibility to participate in the current study and were categorized either as cognitively normal or MCI according to established criteria for diagnosis of MCI^42^,^43^. Neuropsychological tests included Mini Mental State Examination^44^ (MMSE), Montreal Cognitive Assessment^45^ (MOCA), Verbal Learning and Memory Test^46^ (VLMT), Wechsler Memory Scale^47^ (WMS), Boston Naming Test^48^ (BNT) and Trail Making Test A/B^49^. Clinical examination including clinical workup and neuropsychological testing were administered within 30 days of the PiB-PET scan and 7T MRI scan. Isoforms of the APOE gene were assessed for all participants^50^.

Exclusion criteria for the current study were: severe cognitive deficits indicating dementia, significant medication or drug abuse with possible effects on cognition, 7T MRI exclusion criteria (such as history of claustrophobia, vertigo, seizure disorder, middle-ear disorder, double vision and the presence of metals in or on the body), MRI scans with the evidence of infection, infarction, or other focal lesions, clinically relevant changes in red blood cell count, exclusion criteria for PiB-PET, history of severe allergic reactions to drugs or allergens, serious medical or neuropsychiatric illness and significant exposure to radiation.

### Carbon-11 based Pittsburgh compound B Positron Emission Tomography (PiB-PET) for estimation of brain Aβ-plaque density

PiB-PET based estimation was used to estimate individual brain Aβ-plaque-load^40^,^41^. Individual dose of 350 MBq of carbon-labelled PiB was injected into the cubital vein. Standard quantitative filtered back projection algorithm including necessary corrections was applied. Cerebral Aβ deposition values were extracted using PMOD Brain Tool software-package (PNEURO, Version 3.4, PMOD Technologies Ltd, Zürich, Switzerland). Late frame (minutes 50–70) values were standardized by the cerebellar gray matter average, resulting in 3D-volumes of PiB-PET retention (matrix = 128 × 128 × 47, voxel size = 2.3 × 2.3 × 3.3 mm). As a single measure of individual cortical Aβ-plaque-load, cortical PiB retention scores were determined by calculating a composite score using merged cortical PiB-PET intensity values, as reported earlier^51^.

### MRI data acquisition

All subjects were scanned using a Philips 7-Tesla Achieva whole-body scanner (Philips Healthcare, Best, The Netherlands) equipped with a Nova Medical quadrature transmit head coil and 32-channel receive coil array. A T1-weighted MP2RAGE image (TR/TE = 4.8 ms/2.1 ms, voxel size = 0.6 × 0.6 × 0.6 mm^3^, SENSE-factor = 2 × 1 × 2, scan duration = 7:50 min) was acquired for anatomical referencing and automated image segmentation. MR phase measurements used for QSM calculation were acquired using a multi-echo 3D gradient recalled echo (GRE) sequence with 3 echoes (TR/TE/ΔTE = 23/6/6 ms, flip angle = 10°, voxel size = 0.5 × 0.5 × 0.5 mm^3^, SENSE-factor = 2.5 × 1 × 2, flow-compensated, scan duration = 13:48 min). Phase data acquired with an echo time in the range of 12–18 ms was used for QSM reconstruction. rs-fMRI was acquired using 3D T2-prep GRE sequence^38^ (TR = 2s, TR_GRE_/TE_GRE_ = 3.08/1.6 ms, voxel size = 1.5 × 1.5 × 1.5 mm^3^, scan duration = 7:03 min). The high resolution GRE images were inspected (by P.U.) for any imaging artifacts or abnormalities, in particular cerebral microhaemorrhages (microbleeds).

### MRI data processing

#### Quantitative susceptibility mapping (QSM) for measuring brain iron load

Multiple processing steps were performed to calculate from acquired MR phase images the quantitative susceptibility maps of which local cerebral iron load is inferred. First, phase unwrapping was performed using Laplacian based discrete phase unwrapping^26^. A brain mask was then obtained by skull-stripping the GRE magnitude image acquired at TE of 12 ms using FSL's brain extraction tool (BET, FMRIB Oxford, UK) with fractional threshold of 0.3. The unwrapped phase images were then divided by 2π*TE to obtain an image of the frequency shift in Hz for each echo. Subsequently, background fields were eliminated with the sophisticated harmonic artifact reduction for phase data (SHARP)^28^ approach using a variable spherical kernel size with a maximum radius of 4 mm and a regularization parameter of 0.05^28^. After removal of background fields, the resulting images of the two echoes were averaged to obtain a higher SNR as compared to single echo reconstruction^52^. Inverse dipole calculations to obtain the susceptibility maps were performed using a LSQR based minimization^26^,^53^. From suitable reference regions such as white matter tracts and central cerebral spinal fluid (CSF) regions^23^, the region having the lowest standard deviation of mean susceptibility in all subjects was selected. In this sample the frontal central CSF region in the lateral ventricles was selected as a reference region for the final susceptibility quantification. All reported susceptibility values are then relative to the mean susceptibility value of this reference region. Classification of all subjects as “high” or “low” cerebral iron content was performed by a median split of the average cortical gray matter susceptibility of all subjects, in the same regions used for the determination of the individual cortical Aβ-plaque-load^51^.

#### Assessment of structure volumes and mean susceptibility

In order to assess atrophy and susceptibility differences between MCI subjects and controls, the T_1_-weighted image was co-registered to the GRE magnitude image. The co-registered T_1_ image was then segmented using a multi-atlas matching approach developed as part of the Johns Hopkins University brain atlas, which is optimized for the parcellation of non-healthy brains^54^,^55^. ROIs were selected in the basal ganglia and several cortical gray matter structures for which mean susceptibility was calculated after eroding the ROI-masks with two pixels (1 mm) to account for partial volume effects and possible edge artifacts in cortical ROI’s. To normalize different brain sizes across subjects, individual structural volume was corrected with the following approach: *Corrected structure volume = Original structure volume × (group mean intracranial volume/subject intracranial volume)*.

#### fMRI analysis

Pre-processing of the rs-fMRI data was performed using SPM12 (http://www.fil.ion.ucl.ac.uk/spm/), the following steps were performed: realignment, slice time correction, co-registration of structural scan, segmentation, normalization and smoothing (FWHM = 4). The iron classification and MCI status were used as the covariates of interest in connectivity analysis using the CONN toolbox^56^. The signal was filtered with a band-pass filter using the default CONN setting of 0.01–0.1 Hz. Seed-to-voxel analysis was performed with the seed placed in the MPFC. Motion parameters (extracted using the Artifact Detection Tool, ART, https://www.nitrc.org/projects/artifact_detect/), CSF, and white matter were regressed out, as variables of no interest. Connected voxels were included in the mask if they had a False Discovery Rate (FDR) corrected probability of p < 0.001. Using this mask gray matter susceptibility and PiB-PET retention values were extracted and averaged for each subject.

### Statistics

To examine the differences between groups 1-way MANCOVA was performed with the mean magnetic susceptibility or tissue volume of each brain structure as the outcome variable, while controlling for age and gender, followed by False Discovery Rate (FDR) multiple testing correction^57^. Effect sizes were calculated using Cohen’s d. All statistical tests were performed using MATLAB R2014b (Mathworks, Natick, MA).

## Results

### Demographics of the study population

**Figure 1:**
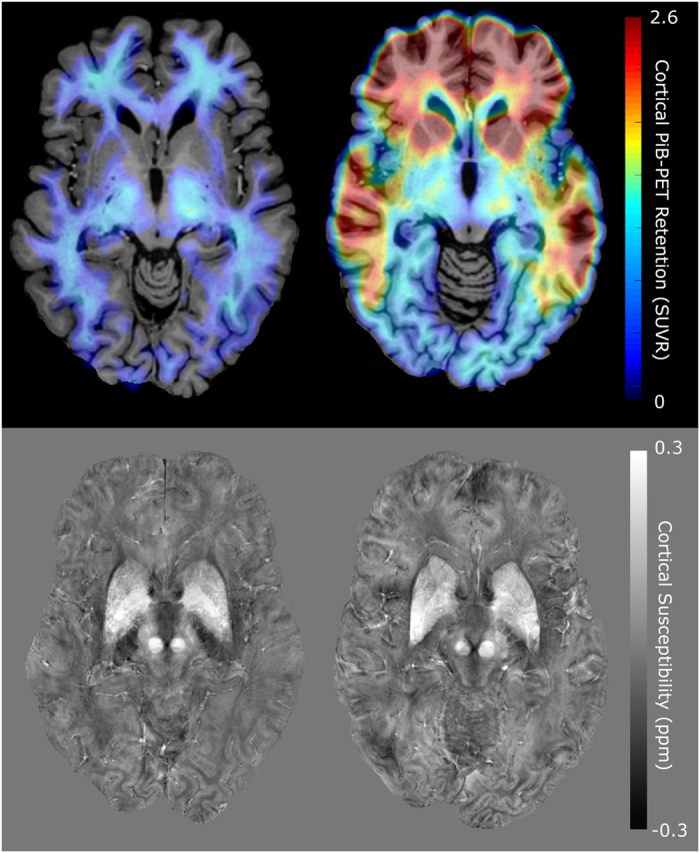
Example images for a control subject (left) and MCI subject (right). The top row shows PiB-PET images of Aβ-plaque-load in gray matter, which is highly increased in the frontal regions in the MCI subject, the signal in the white matter is non-specific to Aβ-plaque-load and is also observed in the control subject. The bottom row shows QSM maps of the same slices indicating regions with high iron load such as the basal ganglia.

Demographic information for the investigated study population and neuropsychological test performance at time of inclusion are summarized in Table 1. MCI and healthy controls differed significantly in scores on the neuropsychological tests MOCA, VMLT, Boston Naming Test and WMS. Example PiB-PET images and QSM maps can be seen in Fig. 1. Cortical PiB-PET retention differed significantly (p = 0.006, effect size = 3.1) between the two groups. The frontal central CSF region in the lateral ventricles, which was used as a reference for susceptibility calculations, was significantly different in volume (healthy: 19.1 ± 2.0 ml, MCI: 24.2 ± 3.3 ml, p < 0.05) but not in absolute susceptibility ppb reading before referencing (healthy: 5.8 ± 1.1 ppb, MCI: 5.6 ± 1.1 ppb). For all subjects, the median split of the average cortical PiB-PET retention was found to be 1.13 and the median split of the average cortical susceptibility was 3.0 ppb. Accordingly, the study population was classified based on PiB-PET retention into “high” and “low” cortical PiB (“high”: 7 healthy, 11 MCI) and susceptibility for iron -load (“high”: 10 healthy, 8 MCI).

### Effects attributable to MCI and APOE-e4 carrier status

Corrected volume was significantly different between controls and MCI subjects in the amygdala, hippocampus, thalamus and putamen with p < 0.001 and effect sizes of 0.80–1.2 (Table 2). However, no significant differences were found for the average susceptibility in any of these regions between the two groups.

Splitting the analysis based on MCI and APOE-e4 status showed no significant susceptibility differences in cortical regions of control subjects but strong significant increases in APOE-e4 carriers in the caudate nucleus (Table 3, p < 0.01, effect size = 1.03) and frontal, temporal, parietal and occipital cortices (p < 0.001, effect sizes = 0.67–1.11) for the MCI group. APOE-e4 positive subjects had significantly higher levels of Aβ-plaque-load in general, as indicated by PiB-PET retention (APOE-e4 positives: 1.56 ± 0.12, APOE-e4 negatives, 1.17 ± 0.04, p = 0.006). There was no significant effect of APOE-e4 status on the volume for any of the investigated cortical and subcortical regions (data not shown).

### The combination of MCI with high iron load is associated with altered MPFC-coupling

**Figure 2:**
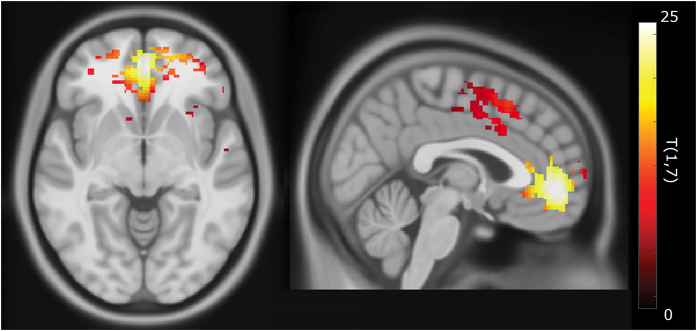
Regions that show significant increased iron associated coupling (T(1,7) above 10.99 indicating p-FDR-corrected <0.001) with the medial prefrontal cortex (MPFC) in subjects with MCI.

The rs-fMRI functional connectivity analyses using MCI status and iron classification as covariates resulted in a mask consisting of 1502 voxels of significantly increased activation (p-FDR-corrected <0.001) with T(1,7) = 10.99. Main regions include frontal pole right (276 voxels, 3% of ROI), paracingulate gyrus left (188 voxels, 14%), frontal medial cortex (145 voxels, 15%), cingulate gyrus (146 voxels, 6%), frontal pole left (122 voxels, 2%) and paracingulate gyrus right (116 voxels, 9%), see also Fig. 2.

### Susceptibility and Aβ-plaque-load correlate within brain regions defined by altered MPFC-coupling

**Figure 3:**
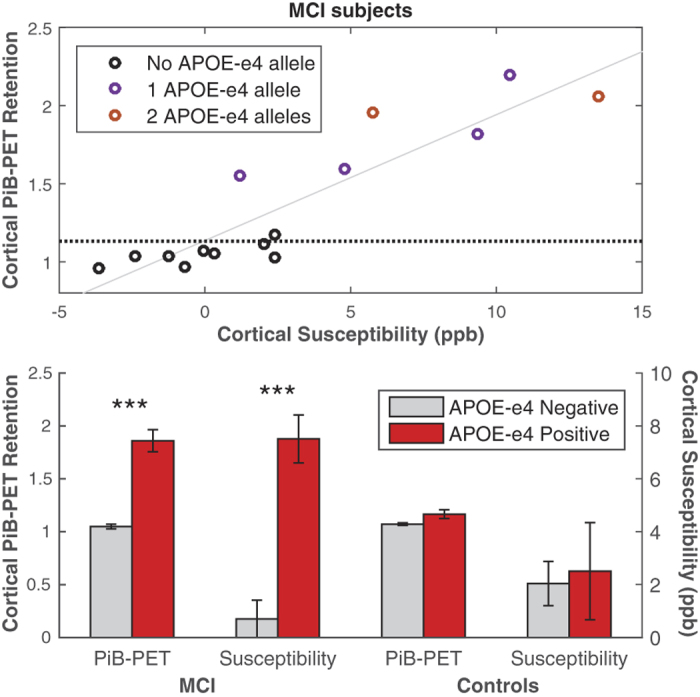
Average Cortical PiB-PET Retention and Cortical Susceptibility of all MCI subjects (top) for the region with significantly increased coupling shown in Fig. 2. The dotted line indicates the median split of the Cortical PiB-PET Retention of all subjects in the study, the trend line is indicated in gray (p < 0.001, Spearman’s rho = 0.86, R^2^-adjusted = 0.80). Average Cortical PiB-PET Retention and Cortical Susceptibility for MCI subjects and controls when split based on APOE-e4 status (bottom), the error bars represent standard error. *** Significant difference between APOE-e4 positive and negative with p < 0.001.

The mask of the region with significantly increased coupling was applied to the individual PiB-PET images and QSM maps of all MCI subjects (Fig. 3). The Spearman’s correlation between cortical PiB-PET retention and susceptibility was found to be p < 0.001 (Spearman’s rho = 0.86, R^2^-adjusted = 0.80). Analysis of the extracted values per group showed significant increases of cortical PiB-PET retention and susceptibility in the APOE-e4 carrier group of the MCI subjects (Fig. 3). Moreover, in the MCI group, the odds ratio for an APOE-e4 carrier to have “high” PiB-PET retention was 48 (p < 0.01, 95% confidence interval = 2.6–932.8) and 17.5 (p < 0.05, 95% confidence interval = 2.2–250.3) to be “high” iron compared to a non-carrier.

## Discussion

In this study magnetic susceptibility was used as a MRI-based measure of cerebral iron load and combined with PiB-PET for measuring Aβ-plaque density in elderly subjects with normal cognition and MCI. For clarity and consistency with earlier studies, changes in susceptibility values will be referred to as changes in iron levels, due to the previously demonstrated correlation of susceptibility values with tissue iron levels in brain gray matter^24^,^27^,^28^,^29^. The main finding of our study is the characterization of brain regions affected by high iron in MCI, within which a spatial colocalization of Aβ-plaques and iron was observable. This effect was associated with increased genetic risk for AD-dementia. As this colocalization is consistent with neuropathologic accounts on AD-signature brain regions^58^, our data may complement earlier considerations on the relationship between cerebral iron and AD-risk^10^. To our knowledge this is the first report on a significant impact of iron on functional network integrity in subjects with MCI.

Although a previous smaller QSM-study reported higher iron load in AD^18^, our data does not show a general effect for MCI when compared to controls (Table 2) indicating such differences might occur in later stages of AD progression. However, MCI subjects with the APOE-e4 allele did show significantly higher iron levels in the neocortex (Table 3), which is a brain region affected by AD-pathology at early stages of disease progression^58^. Our finding of increased cortical iron may therefore support earlier considerations that MCI in APOE-e4 carriers may represent a prodromal stage of AD^6^. Increased cortical iron may be a more specific correlate of emerging neuro-cognitive dysfunction in prodromal AD than cortical Aβ-plaque-load, which was in our data associated with APOE-e4 independently from MCI (Table 3). This may be consistent with earlier data from MRI phase experiments that indicate significant change only for MCI subjects that progressed to dementia^59^ and considerations on synergistic effects of Aβ and other aspects of neurodegeneration in AD^60^,^61^. The fact that reduced volume of subcortical nuclei including the hippocampal area was associated with MCI but not APOE-e4 most likely reflects heterogeneity of possible causes for MCI in the elderly.

It has been shown that the T2-prep BOLD method can achieve comparable contrast-to-noise ratio (CNR) as the conventional echo-planar-imaging (EPI) based BOLD approach, but has much reduced signal dropout and image distortion especially in brain regions close to air cavities such as some frontal and temporal areas^38^. Such dropout and distortion are particularly problematic at 7T where magnetic susceptibility gradients increase substantially at air-tissue boundary. At 3T or lower fields, where most clinical scans are conducted, such EPI artifacts are much reduced. Therefore, the T2-prep BOLD fMRI method in this study at 7T was adopted and it is expected that the findings are generalizable to studies using conventional EPI BOLD fMRI sequences at 3T. For this study a seed based approach investigating functional connectivity of the MPFC was chosen, as the MPFC is a central component of the DMN, which has been demonstrated to be impaired by Aβ pathology already in the preclinical stage of AD^33^. Our findings of iron load being associated with increased coupling in fronto-temporal brain regions is consistent with earlier reports on DMN-change in AD^62^,^63^ and thus indicate that increased iron may contribute to the dysfunction of cognitive brain networks in subjects at risk for AD. However, as the current study investigated combined effects of MCI and iron load for definition of a brain region with particular liability for AD-associated brain change based on altered coupling to the MPFC^64^, our data does not support an independent role of iron for augmenting pathological decline in AD.

The reported correlation (Fig. 3) between cortical iron and Aβ-plaque-load within these functionally altered brain regions suggest that increased cerebral iron relates to regional accumulation of Aβ in subjects at risk for AD and reflects preclinical neuronal dysfunction in AD-signature regions. Additionally, our finding of significantly higher levels of both iron and Aβ in APOE-e4 carriers is consistent with earlier reports and suggests that the APOE-e4 allele may confer susceptibility to AD via brain iron accumulation^10^.

Our data furthermore suggest that the co-occurrence of iron and Aβ may be mediated by APOE-e4, which has been demonstrated to both promote cerebral Aβ accumulation by competing for the same clearance pathways^65^ and increase cerebral iron retention by impaired lipoprotein trafficking due to low affinity of APOE-e4 to high-density lipoprotein^10^. While direct interactions between iron and Aβ may result in increased toxicity by production of redox-active iron forms and oxidative stress^20^,^66^, brain iron accumulation is also associated with microglial over-activation^22^, promoting neurodegeneration in AD^67^. Our observation of altered functional connectivity, may reflect these processes and thus indicate preclinical brain change with the potential of causing progressive neuronal damage, as reflected by worsening neurocognitive disorder. Although the sample size is small, the increased sensitivity at the high field strength of 7T with inherently better SNR in QSM, provides currently the most sensitive detection of *in vivo* gray matter iron levels^29^. When interpreting the current data it needs to be taken into account that the QSM-signal is biased by decreased myelin density^29^,^68^. However, the cortical and deep gray matter regions investigated in this study are low in myelin content and thus the myelin contribution to the susceptibility signal in this study was considered negligible. While spatial co-localization of microhemorrhages with Aβ-plaques may bias iron measures^69^, in the current study no microhemorrhages were observable within the brain regions investigated.

Considering that iron may reflect processes associated with Aβ related neurocognitive dysfunction, further studies are needed to investigate whether the efficacy of therapeutic strategies lowering brain Aβ-plaque-load for slowing down progression of AD^7^,^70^ is affected by the extent of local iron accumulation^11^,^71^.

## Additional Information

**How to cite this article**: van Bergen, J. M. G. *et al*. Colocalization of cerebral iron with Amyloid beta in Mild Cognitive Impairment. *Sci. Rep.* **6**, 35514; doi: 10.1038/srep35514 (2016).

## Figures and Tables

**Table 1:** Demographic data and clinical assessment scores for control subjects with normal cognition and MCI subjects at time of inclusion in the study.

	Controls	MCI
N (F/M)	8/14	5/10
Age (years)	71.91 ± 5.25	75.27 ± 7.63
Education (years)	13.64 ± 2.56	15.2 ± 3.51
PiB-PET retention	1.16 ± 0.08	1.5 ± 0.17**
APOE-e4 positive	7 (31%)	6 (40%)
MMSE	29.27 ± 0.70	28.61 ± 1.65
MOCA	27.36 ± 1.30	24.44 ± 2.17**
VLMT: immediate recall	11.59 ± 2.22	7.11 ± 4.00***
VLMT: delayed recall	11.41 ± 2.94	6.83 ± 4.29***
VLMT: recognition	12.73 ± 2.31	7.78 ± 5.79**
Boston Naming Test	14.68 ± 0.48	13.94 ± 1.26*
WMS: pairs learning	14.76 ± 4.28	10.65 ± 4.72**
WMS: pairs recall	5.52 ± 1.49	3.88 ± 1.75**
Verbal Working Memory	6.32 ± 1.99	5.44 ± 1.42
Trail Making Test ratio	2.58 ± 0.74	2.6 ± 1.24

Data are presented as mean ± standard deviation. APOE-e4 status presented as N (percentage of group). Age and Education are in years. *Significant difference between controls and MCI with p < 0.05, **p < 0.01, ***p < 0.001.

**Table 2:** Changes in corrected volume and susceptibility (referenced to CSF) between controls and MCI.

	Corrected volume (ml) Mean ± STE		χ (ppb) Mean ± STE	
	Controls	MCI	Controls	MCI
Amygdala	4.03 ± 0.09	3.60 ± 0.15***	−16.6 ± 2.3	−17.4 ± 2.3
Nucleus Acc	1.76 ± 0.07	1.84 ± 0.10	11.8 ± 3.7	11.8 ± 5.5
Hippocampus	8.62 ± 0.18	7.55 ± 0.22***	−1.3 ± 1.7	−0.6 ± 2.9
Entorhinal Ctx	2.15 ± 0.13	2.01 ± 0.15	23.4 ± 3.8	25.8 ± 3.3
Thalamus	13.88 ± 0.19	12.83 ± 0.40***	−7.7 ± 1.8	−8.9 ± 1.8
Caudate Nucleus	9.53 ± 0.24	9.31 ± 0.30	41.4 ± 4.2	38.2 ± 3.7
Putamen	10.11 ± 0.27	8.92 ± 0.43***	63.6 ± 4.4	61.2 ± 4.3
Globus Pallidus	3.50 ± 0.07	3.35 ± 0.09	104.5 ± 4.9	99.3 ± 5.0
Frontal Ctx	16.56 ± 1.98	16.13 ± 2.36	2.1 ± 1.8	2.6 ± 2.2
Temporal Ctx	23.24 ± 2.40	22.13 ± 2.77	0.3 ± 1.6	2.1 ± 2.1
Parietal Ctx	21.63 ± 1.13	21.23 ± 1.32	4.3 ± 1.6	4.0 ± 1.8
Occipital Ctx	18.76 ± 2.51	18.38 ± 2.93	3.6 ± 1.8	3.8 ± 2.1

Ctx = Cortex. Data are presented as mean ± standard error (STE). ***Significant difference between controls and MCI with p < 0.001.

**Table 3:** Quantitative magnetic susceptibility (χ in ppb referenced to CSF) and PiB-PET retention (SUVR) separated by APOE-e4 status within the two groups.

	Iron load (χ, ppb) Mean ± STE		*d*	Aβ-plaque-load (PiB-PET, SUVR) Mean ± STE		*d*
	APOE-e4 -	APOE-e4 +		APOE-e4 -	APOE-e4 +	
A) Controls						
Amygdala	−17.2 ± 2.5	−15.3 ± 2.2	0.19	1.19 ± 0.02	1.19 ± 0.02	0.02
Nucleus Acc	12.1 ± 4.1	11.2 ± 3.2	0.06	1.17 ± 0.02	1.23 ± 0.03	0.64*
Hippocampus	−1.4 ± 1.7	−1.2 ± 2.1	0.02	1.25 ± 0.02	1.31 ± 0.02	0.70*
Entorhinal Ctx	22.6 ± 5.1	25.0 ± 2.6	0.15	1.08 ± 0.02	1.14 ± 0.01	0.92**
Thalamus	−6.5 ± 1.9	−10.5 ± 1.4	0.55	1.48 ± 0.04	1.48 ± 0.04	0.01
Caudate Nucleus	46.0 ± 3.7	31.5 ± 5.5	0.75*	1.26 ± 0.03	1.28 ± 0.03	0.23
Putamen	67.2 ± 4.3	55.9 ± 5.3	0.56	1.33 ± 0.02	1.36 ± 0.01	0.46
Globus Pallidus	106.4 ± 5.0	100.4 ± 5.7	0.26	1.45 ± 0.02	1.50 ± 0.04	0.34
Frontal Ctx	2.6 ± 1.9	1.1 ± 2.0	0.18	1.02 ± 0.03	1.13 ± 0.04	0.69***
Temporal Ctx	0.7 ± 1.6	−0.6 ± 2.0	0.17	1.04 ± 0.02	1.10 ± 0.04	0.48***
Parietal Ctx	4.1 ± 1.6	2.7 ± 1.8	0.25	0.99 ± 0.02	1.15 ± 0.05	1.07***
Occipital Ctx	4.0 ± 1.9	2.9 ± 1.8	0.14	1.14 ± 0.03	1.19 ± 0.04	0.42**
B) MCI						
Amygdala	−17.5 ± 1.8	−17.2 ± 2.6	0.04	1.07 ± 0.02	1.54 ± 0.03	4.01***
Nucleus Acc	8.2 ± 4.9	17.3 ± 4.7	0.44	1.16 ± 0.02	2.41 ± 0.07	7.00***
Hippocampus	−2.0 ± 2.0	1.5 ± 3.5	0.31	1.14 ± 0.03	1.42 ± 0.03	2.55***
Entorhinal Ctx	23.1 ± 3.4	30.0 ± 2.5	0.56	1.02 ± 0.03	1.31 ± 0.03	2.80***
Thalamus	−9.1 ± 1.4	−8.5 ± 2.1	0.07	1.46 ± 0.02	1.80 ± 0.06	1.95***
Caudate Nucleus	32.7 ± 2.0	46.3 ± 4.5	1.03**	1.24 ± 0.04	2.13 ± 0.09	3.40***
Putamen	58.6 ± 3.7	65.2 ± 4.0	0.41	1.29 ± 0.01	2.23 ± 0.05	7.29***
Globus Pallidus	96.4 ± 3.3	103.5 ± 6.3	0.36	1.41 ± 0.03	1.92 ± 0.06	2.86***
Frontal Ctx	0.1 ± 1.6	6.3 ± 2.2	0.78***	0.98 ± 0.05	1.95 ± 0.10	3.32***
Temporal Ctx	0.0 ± 1.8	5.2 ± 1.8	0.67***	1.03 ± 0.02	1.73 ± 0.07	3.50***
Parietal Ctx	1.3 ± 1.2	8.1 ± 1.8	1.11***	1.00 ± 0.03	1.87 ± 0.09	3.60***
Occipital Ctx	1.5 ± 1.4	7.2 ± 2.4	0.71***	1.13 ± 0.02	1.59 ± 0.08	2.20***

*Significant difference between APOE-e4 positive and negative with p < 0.05, **p < 0.01, ***p < 0.001. *d* indicates effect sizes (Cohen's *d)*.
